# Imipenem resistance of *Pseudomonas *in pneumonia: a systematic literature review

**DOI:** 10.1186/1471-2466-10-45

**Published:** 2010-08-26

**Authors:** Marya D Zilberberg, Joyce Chen, Samir H Mody, Andrew M Ramsey, Andrew F Shorr

**Affiliations:** 1 School of Public Health and Health Sciences, University of Massachusetts, Amherst, MA, USA; 2 EviMed Research Group, LLC, Goshen, MA, USA; 3 Ortho-McNeil Janssen Scientific Affairs, LLC, Raritan, NJ, USA; 4 Division of Pulmonary and Critical Care Medicine, Washington Hospital Center, Washington, DC, USA

## Abstract

**Background:**

Pneumonia, and particularly nosocomial (NP) and ventilator-associated pneumonias (VAP), results in high morbidity and costs. NPs in particular are likely to be caused by *Pseudomonas aeruginosa *(PA), ~20% of which in observational studies are resistant to imipenem. We sought to identify the burden of PA imipenem resistance in pneumonia.

**Methods:**

We conducted a systematic literature review of randomized controlled trials (RCT) of imipenem treatment for pneumonia published in English between 1993 and 2008. We extracted study, population and treatment characteristics, and proportions caused by PA. Endpoints of interest were: PA resistance to initial antimicrobial treatment, clinical success, microbiologic eradication and on-treatment emergence of resistance of PA.

**Results:**

Of the 46 studies identified, 20 (N = 4,310) included patients with pneumonia (imipenem 1,667, PA 251; comparator 1,661, PA 270). Seven were double blind, and 7 included US data. Comparator arms included a β-lactam (17, [penicillin 6, carbapenem 4, cephalosporin 7, monobactam 1]), aminoglycoside 2, vancomycin 1, and a fluoroquinolone 5; 5 employed double coverage. Thirteen focused exclusively on pneumonia and 7 included pneumonia and other diagnoses. Initial resistance was present in 14.6% (range 4.2-24.0%) of PA isolates in imipenem and 2.5% (range 0.0-7.4%) in comparator groups. Pooled clinical success rates for PA were 45.2% (range 0.0-72.0%) for imipenem and 74.9% (range 0.0-100.0%) for comparator regimens. Microbiologic eradication was achieved in 47.6% (range 0.0%-100.0%) of isolates in the imipenem and 52.8% (range 0.0%-100.0%) in the comparator groups. Resistance emerged in 38.7% (range 5.6-77.8%) PA isolates in imipenem and 21.9% (range 4.8-56.5%) in comparator groups.

**Conclusions:**

In the 15 years of RCTs of imipenem for pneumonia, PA imipenem resistance rates are high, and PA clinical success and microbiologic eradication rates are directionally lower for imipenem than for comparators. Conversely, initial and treatment-emergent resistance is more likely with the imipenem than the comparator regimens.

## Background

Antimicrobial resistance is a growing concern in the US and abroad. Among infections caused by gram-positive pathogens, methicillin-resistant *Staphylococcus aureus *(MRSA) has taken center stage, now accounting for well over 50% of all documented staphylococcal infections in the US [1]. Similarly, hospitalizations with vancomycin-resistant enterococcal (VRE) species are rising rapidly, particularly since 2003 [2,3]. Gram negative bacteria, though not necessarily rising in volume [3], are also becoming increasingly resistant to existing antimicrobials. Most alarming is the evolution of extended-spectrum β-lactamase producing *Enterobacteriaceae*, as well as multidrug resistant *Pseudomonas aeruginosa *(PA), some resistant to multiple drug classes [4,5].

PA in particular is reported to have 20% resistance rates to imipenem, a drug considered to be first-line therapy for ventilator-associated pneumonia, for example, and one that is frequently utilized when the suspicion of PA is high [6]. The patterns of PA resistance are important to appreciate because of the strong evidence that inappropriate empiric therapy leads to increased hospital mortality [7,8], and patients with a resistant infection are less likely to receive appropriate initial treatment [9,10]. For this reason, starting empiric coverage with a broad-spectrum antibiotic followed by de-escalation has been advocated as a strategy to improve outcomes [6]. To justify such a strategy it is important to have complete information on the epidemiology of pathogen resistance, and in the absence of a national surveillance mechanism, all potential sources of such information should be explored. While several primary epidemiologic and microbiology studies have demonstrated reduced imipenem susceptibility among PA [4,5,11,12], the full burden of emerging imipenem resistance reported in the literature has not been quantified. Thus, we performed a systematic review of literature to explore and quantify emerging resistance and reduced susceptibility of PA to imipenem in the setting of pneumonia.

## Methods

We conducted a MEDLINE search using keywords "pneumonia" and "imipenem" with the Boolean operator "AND" joining the two. We restricted this search to papers published in English between January 1993 and December 2008 that were randomized controlled trials, clinical trials, or meta-analyses. Two investigators reviewed all identified studies for relevance and resolved any disagreement by reaching consensus. Because we intended to document emerging trends in the development and characteristics of imipenem resistance among PA isolates, only studies utilizing one of the three specified study designs, including a pharmaceutical comparator, and mentioning both PA and imipenem were included in our analysis.

Two reviewers (AR, MDZ) extracted pertinent data from each study and entered it into data extraction forms developed specifically for the current project. Specifically, we extracted information about geographic location of study population, study period, population characteristics, characterization of therapy, blinding, and factors related to PA. Although our primary interest was PA, we documented the intended primary and secondary endpoints for each study.

Included were data from all patients with at least 1 PA isolate receiving at least 1 dose of the specified treatment. We assumed that patients with PA isolates in studies not specifying a minimum number of antibiotic doses received at least 1 dose and included them in our analysis as well. The outcomes of interest were clinical and microbiological eradication rates for PA among the included patients, as well as initial and emerging resistance of PA to imipenem and comparator drugs, and PA superinfection rates. Wherever possible, we normalized both initial and emerging resistance to the baseline number of patients with PA isolates reported. If it was unclear whether the newly-emergent resistant PA occurred in a patient with a previous PA culture, we assumed that this was the case and utilized the total number of PA isolates reported in the denominator. Each patient was assumed to qualify for one initial or one emergent resistant PA, and thus the words "isolates" and "patients" are used interchangeably throughout this report. Finally, we collected information on adverse outcomes, including development of diarrhea in general and specifically the emergence of *Clostridium difficile *infection (CDI). Studies were excluded if they failed to report at least one of the outcomes of interest.

The success of PA treatment was computed by deriving the proportions of clinical and microbiological eradication across all included studies. We quantified the pooled overall percentage of PA isolates in which resistance was either present at baseline or emerged, as well as the incidence of superinfection. All outcome definitions were those used in the respective primary studies; for example, "clinical success" was defined most frequently as resolution of signs and symptoms related to the infection. We further performed sensitivity analyses in studies incorporating blinding in the experimental protocol, by drug class of the comparator therapy, in studies with nosocomial pneumonia and those performed in North America only. All pooling was performed qualitatively and no attempt at quantitative analyses was made.

## Results

**Figure 1:**
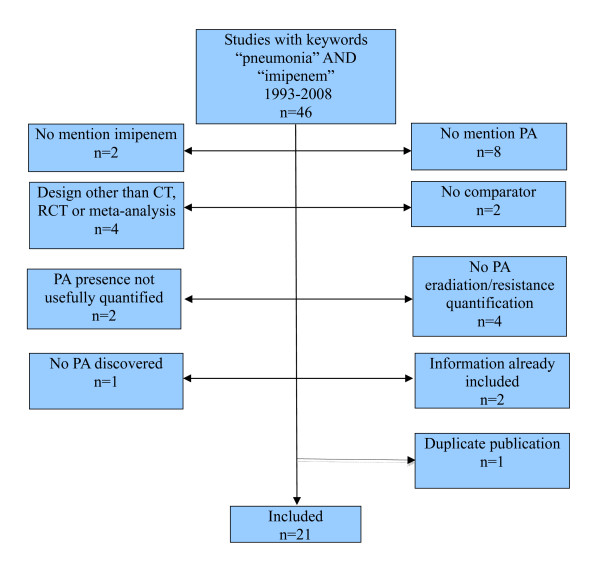
**Study selection flow chart**. PA: *Pseudomonas aeruginosa*; CT: clinical trial; RCT: randomized controlled trial

Of 46 papers identified in our search published between 1993 and 2008, 26 [13-38] were excluded while 20 [39-58] met the inclusion criteria. The reasons for exclusion [13-38] were as follows (Figure 1): no mention of imipenem (n = 2) [13,14]; study designs other than controlled trials, clinical trials or meta-analyses (n = 4) [15-18]; failed to mention PA (n = 8) ([9-26]; provided incomplete quantitative information about PA (n = 2) [27,28]; quantified the presence of PA isolates but did not provide useful information about their eradication or emerging resistance (n = 4) [29-32]; found no PA in initial cultures (n = 1) [33]. Additionally, 2 controlled trials assessed the efficacy of alternative imipenem dosages, and were excluded for this reason [34,35]. To avoid double counting, we ultimately excluded both of the meta-analyses [36,37], since all relevant papers examined therein had already been included, as well as one additional primary report [38] which duplicated a previously reported study [57]. One exception was made to include the study by Shorr and coworkers [44], which is a subgroup analysis of VAP patients enrolled into another study of NP in our study set [46]. This was done for the following reasons: 1). Shorr et al. [44], but not West and colleagues [46], reported on several outcomes of primary interest (specifically PA resistance emergence) in the current review, 2). Eliminating the Shorr study from the analyses did not alter the results substantively, and 3). Several of the sensitivity analyses used only one or the other of the studies, but not both (see below).

In all, 20 clinical trials comprised the final set [39-58] (Table 1). Comparator drug subcategories included a β-lactam arm in 16 studies [39-46,48-51,53,54,56,58], fluoroquinolone in 5 studies [44,46,47,55,57], aminoglycoside in 2 studies [53,56], and vancomycin in 1 study [52]; 6 employed double coverage [44,45,52-54,56]. The primary outcome for 17 of these 20 studies was clinical success [39-52,54,57,58] and 16 of 20 included a microbiological response assessment for underlying pathogens [39-43,45-48,50,52-54,56-58] (Table 1). Ten of 20 studies provided information about the minimum number of imipenem and comparator doses required for inclusion in analysis, 7 of which included patients if they received at least 1 dose of either imipenem or the comparator [39,41,42,46,47,50,57], and 1 study each excluding patients receiving fewer than 5 [49], 6 [40], and 15 [55] doses (Table 2).

**Table 1:** Study characteristics.

First author, year	Centers	Population	Primary endpoint	Comparator regimen	Minimum doses
Chastre, 2008 [39]	M	VAP	C/M success	Doripenem	1
Yanagihara, 2005 [40]	S	CAP	C/M success	Sulbactam/ampicillin	6
Schmit, 2006 [41]	M	NP	Clinical response	Piperacillin/tazobactam	1
Joshi, 2006 [42]	M	NP	Clinical response	Piperacillin/tazobactam	1
Romanelli, 2006 [43]	M	CAP	C/M success	Meropenem	NR
Shorr, 2005 [44]	M	VAP	Clinical success	Levofloxacin/ceftazidime	NR
Zanetti, 2003 [45]	M	NP	C/M success	Cefepime	NR
West, 2003 [46]	M	NP	C/M success	Levofloxacin/ceftazidime	1
Torres, 2000 [47]	M	VAP	C/M success	Ciprofloxacin	1
Bartoloni, 1999 [48]	M	CAP	Clinical response	Meropenem	
Jaccard, 1998 [49]	M	Mixed	Clinical success	Piperacillin/tazobactam	5
Marra*, 1998 [50]	S	Mixed	C/M success, resistance	Piperacillin/tazobactam	1
Ho, 1997 [51]	NR	CAP	Clinical success	Ceftazidime	NR
Raad, 1996 [52]	S	Mixed	C/M success	Aztreonam/vancomycin	NR
Vic, 1996 [53]	S	Mixed	Bacteriological parameters	Ceftazidime/amikacin	NR
Bohme, 1995 [54]	NR	Mixed	C/M success	Ceftazidime/piperacillin	NR
Siami, 1995 [55]	M	CAP & NP	Safety	Ciprofloxacin	15
Cometta, 1994 [56]	M	Mixed	Tolerance	Imipenem/netilmycin	NR
Fink, 1994 [57]	M	CAP & NP	C/M success	Ciprofloxacin	1
Norrby, 1993 [58]	M	Mixed	C/M success	Ceftazidime	NR

M: multicenter, S: single center, NR: not reported, C/M: clinical/microbiological, VAP: ventilator-associated pneumonia, CAP: community-acquired pneumonia, ICU: intensive care unit, NP: nosocomial pneumonia, NR: not reported, CF: cystic fibrosis, PA: *Pseudomonas aeruginosa*

*Although this study did not explicitly report on PA outcomes, it was included because it was one of the few studies specifically reporting zero incidence of *C. difficile *diarrhea

**Table 2:** Results: *P. aeruginosa *clinical and microbiologic success, baseline and emergent resistance rates*

First author, year	Study arm	PA isolates (n)	Clinical success (%)	Microbiologic eradication (%)	Resistance at baseline (%)	Emergent resistance (%)
Chastre, 2008 [39]	Imipenem	25	42.9	35.7	24.0	52.6
	Comparator	28	80.0	65.0	0.0	35.7
Yanagihara, 2005 [40]	Imipenem	4		50.0		
	Comparator	3		0.0		
Joshi, 2006 [42]	Imipenem	17		70.6		
	Comparator	18		76.5		
Romanelli, 2006 [43]	Imipenem	1	0.0	0.0		
	Comparator	1	0.0	0.0		
Shorr, 2005 [44]	Imipenem	18^†^	72.0			5.6
	Comparator	16	85.0			6.3
Zanetti, 2003 [45]	Imipenem	32			15.6	28.1
	Comparator	27			7.4	11.1
West, 2003 [46]	Imipenem	17^†^	41.2	29.4		
	Comparator	17	64.7	58.8		
Torres, 2000 [47]	Imipenem	12	66.0	25.0		33.0
	Comparator	14	71.0	50.0		7.0
Bartoloni, 1999 [48]	Imipenem	3	66.6	66.7		
	Comparator	1	100.0	100.0		
Jaccard, 1998 [49]	Imipenem	24	50.0		4.2	25.0
	Comparator	21	90.5		0.0	4.8
Ho, 1997 [51]	Imipenem	2	50.0			
	Comparator	3	100.0			
Raad, 1996 [52]	Imipenem	1	0.0			
	Comparator	4	75.0			
Vic, 1996 [53]	Imipenem	1		100.0		
	Comparator	19		100.0		
Bohme, 1995 [54]	Imipenem	3	66.0	66.0		
	Comparator	0	NA	NA		
Siami, 1995 [55]	Imipenem	9				77.8
	Comparator	12				41.7
Cometta, 1994 [56]	Imipenem	24		55.6		33.3
	Comparator	23		50.0		56.5
Fink, 1994 [57]	Imipenem	44		25.0		50.0
	Comparator	47		27.7		28.0
Norrby, 1993 [58]	Imipenem	14	42.1			42.9
	Comparator	16	82.4			6.3
Pooled rates	Imipenem	251	45.2	47.6	14.6	38.7
	Comparator	270	74.9	52.8	2.5	21.9

*Limited to studies reporting at least one of these outcomes; empty cells indicate that the corresponding data were not reported

^†^Although the total pseudomonal isolates in the two arms of each analysis add up to the same number (n = 34), the distribution between the arms is different presumably due to reclassification of a single PA isolate from the levofloxacin to the imipenem arm, which occurred between the West [46] publication and the Shorr [44] analysis.

PA:*Pseudomonas aeruginosa*; NA: not applicable

Seven of 20 studies included patients with mixed infections (pneumonia and other diagnoses) [49,50,52-54,56,58], while 13 of the 20 included only patients with pneumonia [39-48,51,55,57] (Table 1): seven with NP [39,41,42,44-47], with 3 of those 7 focusing on VAP only [39,44,47]; 4 with community-acquired pneumonia (CAP) [40,43,48,51], and two with either CAP or NP [55,57]. Of the 4,310 subjects analyzed in the 20 studies, 3,328 had pneumonia: 1,667 in the imipenem and 1,661 in the comparator groups. Determining accurately the exact counts of NP and VAP among the total pneumonia patient population was not feasible.

**Figure 2:**
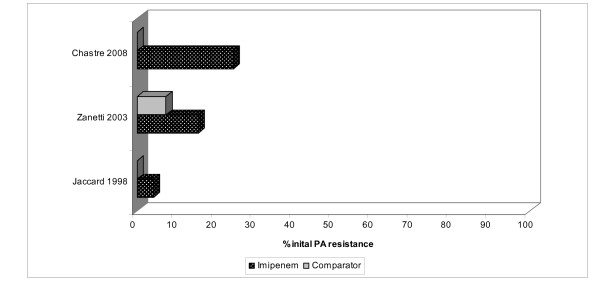
**Proportion of initial reported *P. aeruginosa *resistance to imipenem and comparators graphed chronologically from 1994 to 2009**. PA: *Pseudomonas aeruginosa*

**Figure 3:**
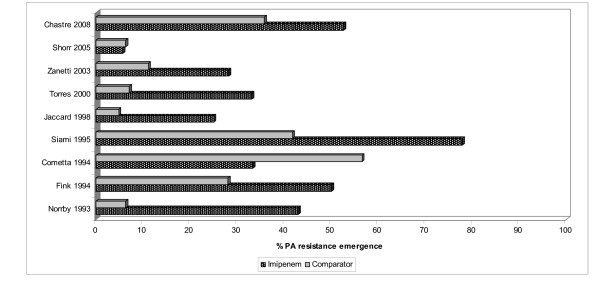
**Proportion of treatment-emergent *P. aeruginosa *resistance to imipenem and comparators graphed chronologically from 1994 to 2009**. PA: *Pseudomonas aeruginosa*

Among the 4,310 subjects, 521 grew out PA isolates in microbiological cultures, 251 in the imipenem and 270 in the comparator groups (Table 2). In 11 studies providing information about clinical PA outcomes [39,43,44,46-49,51,52,54,58], 54 of 120 patients in the imipenem group and 91 of 121 patients in the comparator group were considered clinically cured, with the pooled success rate of 45.2% (range 0.0%-72.0%) for the imipenem and 74.9% (0.0%-100.0%) for the comparator arms. Similarly, in 11 studies providing information on PA microbiological outcomes [39,40,42,43,46-48,53,54,56,57], 72 of 151, 47.6% (range 0.0%-100.0%), of isolates in the imipenem group were successfully eradicated, while 90 of 171, 52.8% (range 0.0%-100.0%), of isolates in the comparator group were eliminated. Three studies [39,45,49] reporting initial PA treatment sensitivities found 12 of 81, 14.6% (range 4.2%-24.0%) of isolates in the imipenem arm and 2 of 76, 2.5% (range 0.0%-7.4%) of isolates in the comparator that were resistant to the initial treatment. In 9 studies reporting the emergence of resistance to the initial antimicrobial among PA isolates [39,44,45,47,49,55-58], resistance to imipenem developed in 78 of 202, 38.7% (range 5.6%-77.8%) instances in the imipenem group and 45 of 204, 21.9% (range 4.8%-56.5%) in the comparator treatment group. Data on the rates of PA superinfection could not be collected reliably. Neither information on average duration of therapy prior to resistance nor the minimum inhibitory concentration (MIC) for imipenem was reported consistently. Although there appeared to be a directional increase in baseline resistance rate of PA to imipenem over time (Figure 2), examining treatment-emergent resistance rates over time did not reveal any clear directional trends (Figures 3).

While adverse events were reported to some extent in nearly every study, the attribution of causation to the respective treatment was rare. Nine of the 21 included studies reported emergence of diarrhea as an adverse event in 104 of 1,576 patients in the imipenem group, 6.6% (range 2.7%-18.1%), and 145 of 1,563 patients in comparator group, 9.3% (range 1.0%-29.3%) [39,41,42,45,46,49,50,52,58]. Only 3 of these 9 studies reported CDI emergence, which was noted in 6 of 334 (1.8%, range 0.0%-5.9%) imipenem-treated patients and 2 of 335 (0.6%, range 0.0%-1.1%) comparator-group patients [45,50,52].

### Sensitivity analyses

#### Blinding

Seven of 20 studies incorporated blinding, 4 of 7 using double-blinding [42,50,55,57] and 3 using investigator-blinding only [41,52,58] (Table 3). Only 2 of these 7 studies [42,57] reported microbiologic eradication of PA, with the aggregate rate of 29 of 61, 47.8% (range 25.0%-70.6%) and 34 of 65, 52.1% (range 27.7%-76.5%) in the imipenem and comparator groups, respectively [42,57]. Further, only 2 of the 7 blinded studies [52,58] provided information on PA clinical success, where the rates of this outcome were 3 of 15, 21.1% (range 0.0%-42.1%), in the imipenem group and 16 of 20, 78.7% (range 75.0%-82.4%), in the comparator group. While none of the studies in this group reported baseline resistance rates, in 3 blinded studies reporting emerging resistance [55,57,58], the rates for this outcome were 38 of 67, 56.9% (range 42.9%-77.8%) and 19 of 75, 25.3% (range 6.3%-41.7%), of imipenem and comparator group PA isolates, respectively.

**Table 3:** Sensitivity analyses*

Subgroup	Study arm	Clinical success		Microbiologic eradication		Resistance at baseline		Emergent resistance	
		PA isolates (n)	% (range)	PA isolates (n)	% (range)	PA isolates (n)	% (range)	PA isolates (n)	% (range)
Blinding present	I	15	21.1 (0.0-42.1)	61	47.8 (25.0-70.6)	NR		67	56.9 (42.9-77.8)
	C	20	78.7 (75.0-82.4)	65	52.1 (27.7-76.5)	NR		75	25.3 (6.3-41.7)
		[52,58]		[42,57]				[55,57,58]	
Comparator drug class									
β-lactam	I	108	39.9 (0.0-72.0)	95	52.7 (0.0-70.6)	81	14.6 (4.2-24.0)	137	31.3 (5.6-52.6)
	C	107	84.7 (0.0-100.0)	110	56.3 (0.0-100.0)	76	2.5 (0.0-7.4)	131	20.1 (4.8-56.5)
		[39,43,44,46,48,49,51,52,54,56]		[39,40,42,43,46,48,53,54,56]		[39,45,49]		[39,44,45,49,56,58]	
Fluoro-quinolone	I	47	59.7 (41.2-72.0)	95	26.5 (25.0-29.4)	NR		83	41.6 (5.6-77.8)
	C	47	73.6 (64.7-85.0)	110	45.5 (27.7-58.8)	NR		89	20.7 (6.3-41.7)
		[44,46,47]		[46,47,57]				[44,47,55,57]	
Nosocomial pneumonia	I	72	55.5 (41.2-72.0)	71	40.2 (25.0-50.0)	57	19.9 (4.2-24.0)	87	29.8 (5.6-52.6)
	C	75	75.2 (64.7-85.0)	77	62.6 (50.0-76.5)	55	3.7 (0.0-7.4)	85	15.0 (6.3-35.7)
		[39,44,46,47]		[39,42,46,47]		[39,45]		[39,44,45,47]	
VAP subgroup	I		60.3 (41.2-72.0)		30.4 (25.0-35.7)	25	24.0 (NA)	55	30.4 (5.6-52.6)
	C		78.7 (71.0-85.0)		57.5 (50.0-65.0)	28	0.0 (NA)	58	16.3 (6.3-35.7)
		[39,44,47]		[39,46]		[39]		[39,44,47]	
North America only	I	36	37.7 (0.0-72.0)	78	41.7 (25.0-70.6)	NR		71	44.4 (5.6-77.8)
	C	37	74.9 (64.0-85.0)	82	54.3 (27.7-76.5)	NR		75	25.3 (6.3-41.7)
		[44,46,52]		[42,46,57]				[44,55,57]	

*Numbers in brackets represent corresponding references

PA: *Pseudomonas aeruginosa*, I: imipenem, C: comparator, NR: not reported, NA: not applicable, VAP: ventilator-associated pneumonia

#### Comparator classes

PA microbiological eradication rates in studies stratified based on antibiotic class of the comparator drug mirrored overall PA eradication rates (Table 3). The pooled rates of clinical success in trials utilizing beta-lactams and reporting this outcome [39,43,44,46,48,49,51,52,54,56] were 39.9% (range 0.0%-72.0%) and 84.7% (0.0%-100.0%) in the imipenem and comparator groups, with corresponding microbiologic eradication rates [39,40,42,43,46,48,53,54,56] of 52.7% (range 0.0% - 70.6%) for imipenem and 56.3% (range 0.0%-100.0%) for comparators. Three beta-lactam studies reported initial resistance rates [39,45,49], and they were 14.6% (range 4.2%-24.0%) and 2.5% (range 0.0% - 7.4%) for imipenem and comparator groups, respectively. Resistance emergence was reported in 6 studies [39,44,45,49,56,58] in 31.3% (range 5.6%-52.6%) of imipenem and 20.1% (range 4.8%-56.5%) of comparator group PA isolates. A pooled analysis of the subgroup of beta-lactam trials specifically comparing imipenem to another carbapenem revealed clinical success rates of 36.5% (range 0.0%-66.7%) in the imipenem and 60.0% (range 0.0%-100.0%) in the comparator carbapenem groups [39,43,48], while the microbiologic eradication rates were 39.5% (range 0.0%-66.7%) for imipenem and 53.8% (range 0.0%-100.0%) for the comparator carbapenems [39,43,48,56]. Only one carbapenem study reported initial resistance, which was detected in 24.0% of patients in the imipenem and 0.0% in the doripenem groups [39]. In the same study comparing doripenem to imipenem in patients with VAP, the rate of resistance emergence was 52.6% in the imipenem and 35.7% in the doripenem groups [39]. This is the only study where resistance emergence was defined explicitly, and denoted a decrease in susceptibility being a 4+-fold increase in the MIC [39]. Notably true resistance (defined as MIC ≥ 8 μg/mL) developed in 20% in the doripenem arm and 57% in the imipenem arm [39]. The only other carbapenem study reporting emergence of resistance noted this rate to be 33.3% in the imipenem and 56.5% in the meropenem groups [56]. Finally, in the studies comparing imipenem to treatment with a fluoroquinolone, imipenem demonstrated a clinical success rate of 59.7% (range 41.2%-72.0%), while that for the comparators was 73.6% (range 64.7%-85.0%) [44,46,47]. Microbiologic eradication rates were 26.5% (range 25.0%-29.4%) for imipenem compared with 45.5% (range 27.7%-58.8%) in the comparator groups [46,47,57]. While baseline resistance rates were not reported in any of the fluoroquinolone studies, resistance emerged at the rates of 41.6% (range 5.6%-77.8%) in the imipenem and 20.7% (range 6.3%-41.7%) in the comparator fluoroquinolone groups [44,47,55,57].

#### Nosocomial pneumonia

In 7 studies enrolling exclusively NP patients, the results were similar to the overall findings [39,41,42,44-47] (Table 3). Clinical success in the imipenem arms was 55.5% (range 41.2%-72.0%) and in the comparator arms 75.2% (range 64.7%-85.0%) [39,44,46,47]. Microbiologic eradication rates were 40.2% (range 25.0%-50.0%) in the imipenem and 62.6% (range 50.0%-76.5%) in the comparator groups [39,42,46,47]. In the 2 studies reporting baseline resistance this rate was 19.9% (range 4.2%-24.0%) in the imipenem and 3.7% (range 0.0%-7.4%) in the comparator groups [39,45]. Four studies in this subgroup reported resistance emergence, and these pooled rates were 29.8% (range 5.6%-52.6%) in the imipenem and 15.0% (range 6.3%-35.7%) in the comparator groups [39,44,45,47]. A further look at VAP patients only revealed similar rates of 60.3% (range 41.2%-72.0%) and 78.7% (range 71.0%-85.0%) imipenem and comparator clinical success rates, respectively [39,44,47], while the corresponding microbiologic eradication rates for imipenem and comparators were 30.4% (range 25.0%-35.7%) and 57.5% (range 50.0%-65.0%) [39,46].

#### Geographic location

The final sensitivity analysis was confined to the studies conducted only in North America [42,44,46,50,52,55,57]. In the 3 studies in this group reporting clinical success, the pooled rates of clinical success were 37.7% (range 0.0%-72.0%) and 74.9% (range 64.0%-85.0%) for the imipenem and comparator groups, respectively [44,46,52]. The corresponding microbiologic eradication rates were 41.7% (range 25.0%-70.6%) in the imipenem and 54.3% (range 27.7%-76.5%) in the comparator groups [42,46,57]. While none of the North American studies reported baseline resistance rates, resistance emerged in 44.4% (range 5.6%-77.8%) of imipenem and 25.3% (range 6.3%-41.7%) of comparator group isolates [44,55,57].

## Discussion

The current systematic review confirms clinical experience that many PA isolates are likely to be resistant to imipenem at the initiation of treatment and, importantly, are likely to develop treatment-emergent resistance. Not only does PA account for 12% of all reported pathogens in pneumonia, 14.6% of all PA isolates exhibit resistance to imipenem at the initiation of treatment, and an additional 38.7% develop this during the course of treatment with imipenem. For comparator interventions, these rates are directionally lower, albeit still substantial, with 2.5% initial and 21.9% emergent resistance. While directionally slightly higher for both imipenem and comparator arms in the trials employing blinding, these general rates persisted across all sensitivity analyses.

As antibiotic resistance continues to escalate among both gram-positive and gram-negative pathogens [1,3-5], for PA specifically, a recent survey from the National Nosocomial Infections Surveillance Network [4] reported year 2003 PA resistance rates of approximately 32%, 20% and 30% to third generation cephalosporins, imipenem and quinolones, respectively, representing 20%, 15% and 9% growth, respectively, over the average resistance rates observed between 1998 and 2002 [4]. The importance of this development cannot be overstated for several reasons. First, it is clear that, similar to other resistant infections, multidrug resistant PA confers worsenes hospital outcomes, including increased mortality, prolonged length of stay and a rise in costs [59-61]. Second, and key to these outcomes at least in part, is the fact that a patient infected with a resistant pathogen is far more likely to be treated with an inappropriate initial antibiotic (one to which the isolate does not exhibit sensitivity in vitro) than with an appropriate one, a choice that approximately doubles the patient's risk of death [9,10]. Third, the rapid evolution of antimicrobial resistance is outpacing efforts to develop and manufacture newer therapies that are effective against new pathogens [62,63]. For all these reasons, and most importantly to improve patient-level outcomes, the patterns of antimicrobial resistance remain critical to study, and this current effort adds to the epidemiologic and microbiology-based knowledge of the burden of PA resistance to imipenem.

The rates of imipenem resistance among PA we uncovered were higher than the 20% reported by the NNIS in year 2003; ours approached 15% at baseline and developed on treatment in a further 39% of the isolates [4]. The differences between the two sources most likely represent differences in populations, in case definitions and in sampling methods. Although neither source is completely generalizable to real-world practice, (trials usually represent a highly select population and the composition of the NNIS hospitals is not disclosed and may not be representative of the US institutions overall) both sources confirm that the problem is grave. Interestingly, similar to the NNIS data, we observed an increase in the prevalence of baseline, but not emergent Pseudomonal imipenem resistance over time (Figure 1).

Our study has additionally documented substantial rates of emergent PA resistance while on treatment, particularly with imipenem. This result echoes the data reported in the meta-analysis by Siempos and colleagues, where development of resistance by PA during treatment for nosocomial pneumonia was higher in patients on carbapenems (mainly imipenem) than other antimicrobials, and that carbapenems were associated with lower treatment success when compared to other antimicrobials [64].

Aside from lending credence to clinical gestalt and confirming epidemiologic observations, our study further suggested that there is an opportunity to use ongoing pooled analyses of clinical data to understand antibiotic resistance issue as an adverse event. Because no individual study is likely to be powered to detect significant rates of baseline or emergent resistance, pharmacoepidemiologic surveillance methods should be advocated to quantify significant trends in this outcome. These techniques can be used as validation strategies for data obtained in epidemiologic and microbiology-based studies. Furthermore, our data point to the need to use integrated databases that include pharmacy and laboratory data as well for ongoing monitoring of antimicrobial resistance among specific organisms, such as PA.

Our study had a number of limitations, most of them driven by the limitations of either design or reporting of the primary trials. First, not all studies allowed us to extract data pertaining only to pneumonia patients, and therefore a small proportion of the overall aggregate results does not pertain to this disease. To counterbalance this issue, we performed a sensitivity analysis among patients with only nosocomial pneumonia, and the results in this group were not substantively different from those in the overall population. Second, despite the aggregate sample size of over 4,000 subjects with pneumonia, the subgroup growing out Pseudomonal pathogens was small, accounting for 12% of all cases. Furthermore, since not all endpoints of interest were reported in every study, the number of PA isolates continued to drift lower in specific analyses, lending limited power for drawing firm conclusions. For this reason, and due to inherent limitation of the data (i.e., PA was never the primary focus of the study) we did not attempt to perform statistical testing. Nevertheless, the fact that most sensitivity analyses resulted in similar proportions of resistance detection lends further credibility to the numbers. It is worth underscoring that some of the ranges of clinical and microbiologic response rates went from 0.0% to 100.0% (Tables 2, 3), the width of the range ostensibly reducing the usefulness of our observation. However, we must point out that four of the five studies reporting these rates each included only one pseudomonal isolate [43,48,51-53], making these estimates neither clinically nor statistically meaningful. Excluding these rates from the clinical success endpoint, for example, would result in the range from 41.2% to 72.0% in the imipenem and from 64.7% to 90.5% for the comparator groups (Table 2). Third, only a handful of the trials employed blinding to reduce bias, and in those trials the resistance rates were directionally slightly higher than in ones without blinding. Despite these limitations, this body of evidence further served to call attention to the alarming rates of antimicrobial resistance among PA in general, and to imipenem specifically.

## Conclusions

In summary, pooling observations from clinical trials occurring over a 15-year period we computed the cumulative PA resistance rate to imipenem to be in the range of 50%, persisting in many sensitivity analyses. The 15% resistance rate at baseline further stresses the importance of risk stratification at bedside and the need to employ early appropriate therapy to improve patient outcomes. The additional 39% resistance development on treatment is also sobering, implying that 1) clinicians employing early broad spectrum coverage need to pay exquisite attention to prompt de-escalation if such coverage is not needed, and 2) researchers need to focus on developing novel therapies whose mechanisms of action may be less subject to the microbial adaptation apparatus. In all, our data add to the epidemiologic alarm of PA resistance and help further sound the call to arrive at strategies that balance patient outcomes with the growing public health threat of antimicrobial resistance.

## Competing interests

This study was funded by a grant from Ortho-McNeil Janssen Scientific Affairs, LLC, Raritan, NJ, USA, the manufacturer of doripenem.

## Authors' contributions

MDZ contributed to study conception and design, and analysis and interpretation of data; was involved in drafting the manuscript and revising it critically for important intellectual content; and gave final approval of the version to be published. JC contributed to study conception and design and acquisition of data; was involved in revising the manuscript critically for important intellectual content; and gave final approval of the version to be published. SHM contributed to conception and design, and acquisition of data; was involved in revising the manuscript critically for important intellectual content; and gave final approval of the version to be published. AMR made substantial contributions to conception and design, acquisition and interpretation of data; was involved in drafting the manuscript; and gave final approval of the version to be published. AFS have made substantial contributions to conception and design, analysis and interpretation of data; was involved in revising the manuscript critically for important intellectual content; and gave final approval of the version to be published.

## Pre-publication history

The pre-publication history for this paper can be accessed here:

http://www.biomedcentral.com/1471-2466/10/45/prepub
